# The Milk of Cows Immunized with Trivalent Inactivated Vaccines Provides Broad-Spectrum Passive Protection against Hand, Foot, and Mouth Disease in Neonatal Mice

**DOI:** 10.3390/vaccines12060570

**Published:** 2024-05-23

**Authors:** Xiaohui Wei, Jing Wu, Wanjun Peng, Xin Chen, Lihong Zhang, Na Rong, Hekai Yang, Gengxin Zhang, Gaoying Zhang, Binbin Zhao, Jiangning Liu

**Affiliations:** 1 NHC Key Laboratory of Human Disease Comparative Medicine, Beijing Key Laboratory for Animal Models of Emerging and Reemerging Infectious Diseases, Institute of Laboratory Animal Science, Chinese Academy of Medical Sciences and Comparative Medicine Center, Peking Union Medical College, Beijing 100021, China; xiaohui-wei@foxmail.com (X.W.);; 2 Wuhan Servicebio Technology Co., Ltd., Wuhan 430079, China; gaoyingzhang@servicebio.cn

**Keywords:** HFMD, maternal antibody, passive immune protection, multivalent antiviral milk, neonatal mouse challenge model

## Abstract

Hand, foot, and mouth disease (HFMD) is a contagious viral infection predominantly affecting infants and young children, caused by multiple enteroviruses, including Enterovirus 71 (EV71), Coxsackievirus A16 (CA16), Coxsackievirus A10 (CA10), and Coxsackievirus A6 (CA6). The high pathogenicity of HFMD has garnered significant attention. Currently, there is no specific treatment or broad-spectrum preventive measure available for HFMD, and existing monovalent vaccines have limited impact on the overall incidence or prevalence of the disease. Consequently, with the emergence of new viral strains driven by vaccine pressure, there is an urgent need to develop strategies for the rapid response and control of new outbreaks. In this study, we demonstrated the broad protective effect of maternal antibodies against three types of HFMD by immunizing mother mice with a trivalent inactivated vaccine targeting EV71, CA16, and CA10, using a neonatal mouse challenge model. Based on the feasibility of maternal antibodies as a form of passive immunization to prevent HFMD, we prepared a multivalent antiviral milk by immunizing dairy cows with the trivalent inactivated vaccine to target multiple HFMD viruses. In the neonatal mouse challenge model, this immunized milk exhibited extensive passive protection against oral infections caused by the three HFMD viruses. Compared to vaccines, this strategy may offer a rapid and broadly applicable approach to providing passive immunity for the prevention of HFMD, particularly in response to the swift emergence and spread of new variants.

## 1. Introduction

Hand, foot, and mouth disease (HFMD) is a global infectious disease in infants and young children caused by various enteroviruses, which can cause fever, blisters, as well as serious complications such as meningitis, encephalitis, acute flaccid paralysis, neurorespiratory syndrome, long-term neurological sequelae, and in the most severe cases, fatal outcomes [1,2,3,4,5,6]. In China’s surveillance report in 2021, the incidence of HFMD exceeded 1.35 million cases, yielding an incidence rate of 96.08 per 100,000 individuals, ranking first among all known infectious diseases [7]. Historically, the mortality rate associated with HFMD has reached levels as high as 1.8%, thereby constituting a major public health problem [8,9]. To date, there remains a notable absence of any approved therapeutic interventions or broad-spectrum protective vaccines targeting HFMD [10,11]. Although the EV71-inactivated vaccine has shown a protection rate exceeding 90% against EV71 [12,13,14,15], its scope of protection remains constrained, as it fails to offer effective prevention against other HFMD-causing viruses such as CA16, CA10, and CA6, among others [10,16,17,18,19]. It is noteworthy that the widespread implementation of this vaccine has indeed yielded a substantial reduction in cases of EV71-related HFMD, especially severe cases [14,20,21,22]. However, it appears to have had a more limited impact on the overall prevalence and incidence rate of HFMD as a whole [20,23]. Moreover, children with a low vaccine response, even after infection with HFMD, may not be able to induce a sufficient immune response and are prone to reinfection with an enterovirus of the same serotype [24,25,26,27]. Therefore, there is a compelling imperative to pursue the development of broad-spectrum vaccines capable of affording more comprehensive protection against HFMD [28,29]. However, as there are 36 kinds of serotypes of enteroviruses that could cause HFMD, it can be predicted that the continuous development of new vaccines targeting prevalent pathogens will not stop. Consequently, the ongoing dynamism of pathogenic variation renders conventional vaccine development and vaccination strategies insufficient in their capacity to counteract this ever-shifting landscape [30,31,32,33,34].

Passive immunity may be a reasonable and effective transitional approach for HFMD prevention in infants. Studies have been reported that the incidence rate of HFMD peaks at the age of one year and decreases with age [35,36]. The average incidence rate among children aged from six to eleven months (31.9 per 1000 people per year) is significantly higher than that among children under five months (2.6 per 1000 people per year), which may be attributed to protection via passive maternal immunity [36]. Similar studies have also shown that approximately 50% of newborn infants have significant levels of anti-EV71 antibodies that were acquired from the mother through placental transmission and breastfeeding, and the protective effect of maternal antibodies began to significantly decline after six months [37]. In addition to HFMD, maternal immunity bestows protective immunity to safeguard infants against a spectrum of other pathogens, including influenza and COVID-19 [38]. During the development of a multivalent vaccine for HFMD, it was also found that immunization with the multivalent vaccine could confer broad-spectrum passive protection to newborn mice [39]. Another study also demonstrated that sera from immunized subjects could provide protective passive immunity to recipient mice. Therefore, passive immunization may be an effective strategy for preventing HFMD [40].

In this study, we used trivalent inactivated EV71, CA16, and CA10 viruses to immunize maternal mice and once again confirmed that maternal antibodies provide broad protective passive immunity against HFMD to their newborns. Building on these findings, we further proposed a multivalent milk targeting various HFMD pathogens and demonstrated its broad-spectrum passive protective effect on newborn mice. Although milk and serum have been proven successful in cross-species passive immunity, research specifically targeting HFMD remains limited [41,42,43,44]. Given the risks associated with cross-species serum use in humans, milk containing neutralizing antibodies against multiple enteroviruses presents a unique active advantage. Therefore, the multivalent milk studied here shows great potential in HFMD prevention. Despite the long path ahead for the clinical implementation of this strategy, considering the rapid mutation rate of the virus, it could serve as a supplementary measure, allowing for quick responses to emerging epidemics before widespread immunization through vaccination.

## 2. Materials and Methods

### 2.1. Viruses and Cell Lines

The HFMD strain information used in this study is shown in Table S1. The virus was subcultured in rhabdomyosarcoma (RD) cells and titrated. In brief, the virus was propagated in RD cells grown in Dulbecco’s minimal essential medium (DMEM) supplemented with 10% (*v*/*v*) heat-inactivated fetal bovine serum (FBS) and 1% penicillin–streptomycin. To determine a 50% tissue culture infective dose (TCID_50_), culture media containing the virus was continuously inoculated into RD cells in a 10-fold dilution. After 3 days of incubation, the endpoint dilution was calculated by the cytopathic effects (CPEs), which were only observed in ½ of the replicate cultures. The Reed–Muench endpoint method was used to calculate the virus titer.

The inactivated virus was prepared by RD cell culture, and the cell supernatant was initially purified by ultracentrifugation and concentrated through ultrafiltration membrane to obtain the virus concentrate (1 × 10^6^ TCID_50_/mL). Each virus concentrate was included in the vaccine cocktail at the same titer.

### 2.2. Animal Models

All specific pathogen-free (SPF) BALB/c mice were obtained from Beijing HFK Bioscience Ltd. In maternal antibody protection experiments, EV71, CA16, CA10, and a mixture of three viruses were inactivated and then emulsified with Freund’s adjuvant for immunization in eight-week-old BALB/c mice [45,46]. The inactivated virus was administered via intramuscular injection into the limbs and immunized at a rate of 100 μL per individual. The first immunization was emulsified with Freund’s complete adjuvant, while the second immunization was emulsified with Freund’s incomplete adjuvant. The interval between the primary injection and booster was 14 days, and serum samples were collected for antibody determination at 14 days, 28 days, and 180 days post the first immunization. After the booster vaccination, female and male mice mated in cages and gave birth to neonatal mice that were then subjected to viral challenge. Briefly, multiple groups of one-day-old neonatal mice were individually infected with 100 × 50% lethal dose (LD_50_) EV71, CA16, and CA10 viruses by gavage of 10 μL virus solution. The weight, mortality, and clinical symptoms of the neonatal mice were continuously recorded; the mice were euthanized; and tissue samples were taken at 5 days post-infection (dpi). Clinical symptoms were graded as shown in Table S2. The collected tissues were used for subsequent virological and pathological assessment.

Six pregnant cows, aged 3–5 years, were purchased from Beijing Kerun Weide Biotechnology Co., Ltd. (Beijing, China) and confirmed to be negative for foot and mouth disease virus types O, A, and Asia 1 by the PrioCHECK FMDV Antibody ELISA Kit (Thermo Fisher Scientific, Waltham, MA, USA, 7610420/7610850/7610870). In the antiviral protection study of milk, the HFMD trivalent inactivated virus emulsified with Freund’s adjuvant was separately administered to three expectant cows at a dose of 10 mL (1 × 10^6^ TCID_50_ per virus) per cow for the first immunization, and the booster was given 14 days later. The first immunization was emulsified with Freund’s complete adjuvant, while the second immunization was emulsified with Freund’s incomplete adjuvant. The control group was only immunized with Freund’s adjuvant. Cow’s milk was collected for antibody determination at 0 days, 7 days, 30 days, 90 days, and 180 days postpartum. Additionally, 100 μL of immune colostrum or control colostrum was mixed with EV71, CA16, and CA10 viruses at a concentration of 100 LD_50_ and then used to infect seven-day-old SPF neonatal BALB/c mice by gavage. Subsequently, continuous monitoring of weight changes and clinical symptoms was carried out on infected young mice, and tissue samples were collected at 5 dpi for virological and pathological assessment.

The animal trials in this study were performed according to the Chinese Regulations for Laboratory Animal Management, the Guidelines for the Care of Laboratory Animals (Ministry of Science and Technology of the People’s Republic of China) and the Laboratory animal— Requirements of environment and housing facilities [47] (GB14925-2010, National Laboratory Animal Standardization Technical Committee). The license numbers associated with this research protocol are LJN17002, LJN18002, LJN21003 and LJN21005, and this study was approved by the Institutional Animal Care and Use Committee of the Institute of Laboratory Animal Science, Peking Union Medical College.

### 2.3. RT–PCR Assay for Viral RNA Analysis

Total RNA was extracted from mouse blood and tissue using TRIzol reagent (Invitrogen, Waltham, MA, USA), and cDNA was obtained by reverse transcription using the RevertAid First Strand cDNA Synthesis Kit (Thermo Fisher Scientific). The RT–PCR assay for virus RNA detection was performed using a QuantiTect Probe RT–PCR kit (QIAGEN Inc., Hilden, Germany) or TB Green Premix Ex Taq II (Takara, San Jose, CA, USA) according to the manufacturer‘s instructions. A standard curve was generated using a series of 10-fold dilutions of the recombinant plasmid at a known concentration. Primers and probes are listed in Table S3.

### 2.4. Enzyme-Linked Immunosorbent Assay for Antibody Level Determination

Determination of virus-specific antibody levels was performed by indirect enzyme-linked immunosorbent assay (ELISA). In brief, inactivated cell suspension containing CA10, CA16, or EV71 viruses are initially purified by ultracentrifugation and concentrated through ultrafiltration membrane to obtain the virus concentrate. Then, 96-well plates coated with 0.5 μg of cell suspension containing inactivated virus at 4 °C overnight were blocked with 2% BSA/PBST at room temperature for 2 h. Subsequently, 2-fold serial dilutions of the serum and milk samples were prepared. Diluted samples were added to wells and maintained at 37 °C for 45 min, followed by incubation with an antibody labeled with horseradish peroxidase (HRP) (goat anti-mouse IgG H&L, Abcam, Cambridge, UK, Ab6789. Rabbit Anti-Cow IgG H&L, Abcam, Ab6927. Sheep Anti-Cow IgA H&L, Abcam, Ab112755) at 37 °C for 45 min. The reaction was initiated using 3,3′,5,5′-tetramethylbenzidine (TMB) substrate (Thermo Fisher Scientific), and the absorbance was measured at 450 nm. All experiments were performed in duplicate.

The concentrations of IgG, IgA, and IgM antibodies in milk were determined according to the manufacturer’s instructions (Bethyl, E11-118, E11-131, E11-101). In short, multiple dilutions of milk were performed, and the antibody concentrations were calculated by ELISA based on an established standard curve.

### 2.5. Antivirus-Neutralizing Antibody Assay

Serum or milk was heated at 56 °C for 30 min to inactivate complement factors and serially diluted 2-fold from 1:2 to 1:4,096. Diluted milk was incubated with 100 TCID_50_ CA10, CA16, or EV71 for 1 h at 37 °C, and the mixture was then mixed with 1 × 10^5^ RD cells. Each dilution was performed in duplicate. After 3 days of incubation, the endpoint dilution was calculated by the CPE observed in replicate cultures. The virus neutralization (VN) titer was expressed as the log2 of the highest dilution.

### 2.6. Histopathological Assessment

The mice were euthanized, and skeletal muscle was collected. Before further treatment, the tissues were fixed with 40 mL of 10% (*v*/*v*) neutral buffered formalin suspension for 7 days. The tissue was embedded in paraffin, and the sections were stained with hematoxylin and eosin. Tissue sections were visualized using PANORAMIC 1000 (3DHISTECH, Budapest, Hungary) and analyzed using CaseViewer2.4 (3DHISTECH). Based on histopathological analysis, a comprehensive cumulative evaluation was conducted from six aspects, including degeneration, necrosis, inflammatory cell infiltration, proliferation, atrophy, and bleeding. The scoring of tissue pathological damage refers to the standards in Table S4.

### 2.7. Quantification and Statistical Analysis

Data and statistical analyses were completed in GraphPad Prism software version 9. The statistical details of the experiments are provided in the corresponding legend. Data plotted on a linear or logarithmic scale are expressed as the mean ± SEM. Technical and biological replicas are described in the illustration. Statistically significant differences were determined using unpaired *t*-tests and two-way ANOVA. Statistical significance was defined as *p* values < 0.05. * *p* < 0.05, ** *p* < 0.01, *** *p* < 0.001, **** *p* < 0.0001.

## 3. Results

### 3.1. Induction of Broad-Spectrum Antibodies in Mice Immunized with Trivalent Inactivated Viruses

**Figure 1:**
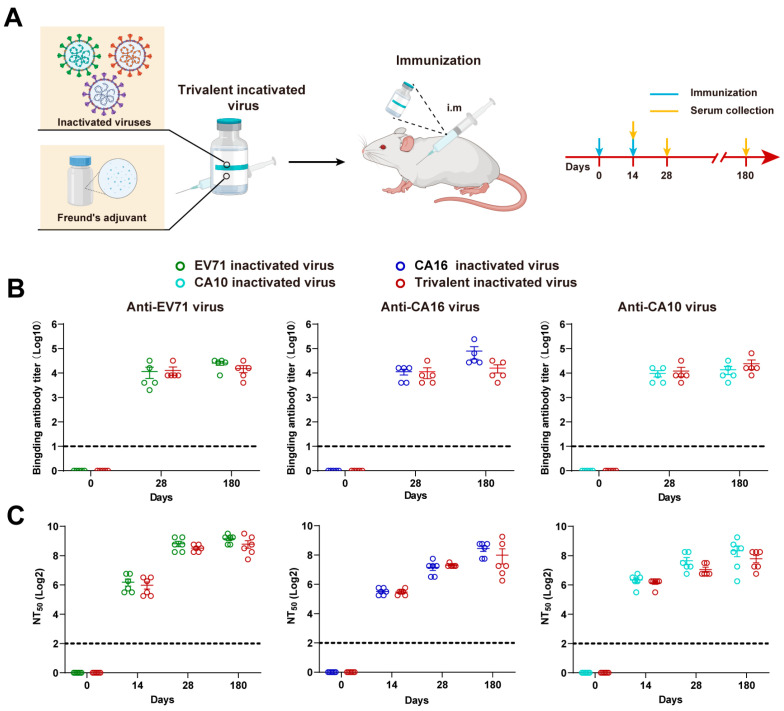
Serum antibody levels in mice vaccinated with HFMD trivalent inactivated viruses. (**A**) Schematic representation of trivalent inactivated virus preparation, mouse immunization, and sampling (*n* = 5–6). (**B**) Quantification of EV71/CA16/CA10 virus-specific antibodies in serum at 28 days and 180 days post immunization with monovalent or trivalent inactivated virus. (**C**) Measurement of EV71/CA16/CA10 virus-neutralizing antibodies in serum at 14 days, 28 days, and 180 days post immunization with monovalent or trivalent inactivated virus.

To reconfirm the protective effect of maternal antibodies and ascertain the feasibility of multivalent HFMD vaccine strategies, we initiated our study by formulating a trivalent inactivated virus comprising the epidemic strains EV71/CA16/CA10 for subsequent immunization in mice and evaluated its immunogenicity and efficacy (Figure 1A). The determination of serum antibodies confirmed that both monovalent and multivalent vaccines can elicit robust immune responses, yielding comparable levels of virus-specific antibodies and neutralizing antibodies. Following a booster immunization, the level of neutralizing antibodies increases. Importantly, the levels of specific antibodies and neutralizing antibodies can be sustained for at least 180 days, as compared to the levels observed at 28 days.

### 3.2. Maternal Antibodies Protected Neonatal Mice from Lethal Damage Caused by Various HFMD Viruses

**Figure 2:**
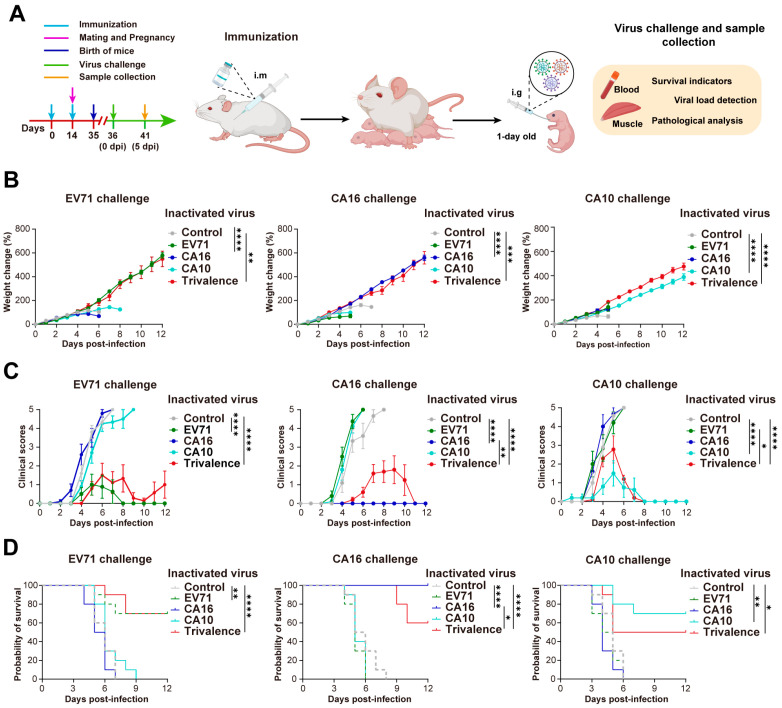
Protective effect of maternal antibodies against HFMD challenge in neonatal mice. (**A**) Experimental design. Eight-week-old female mice were immunized with EV71 (*n* = 12), CA16 (*n* = 12), and CA10 (*n* = 12) monovalent or trivalent (*n* = 12) inactivated virus. Offspring mice were challenged one day after birth (*n* = 48/group). (**B**) Weight changes in one-day-old neonatal mice infected with EV71/CA16/CA10 virus. (**C**) Clinical symptom scores of neonatal mice. (**D**) Survival rate of neonatal mice post EV71, CA10, or CA16 challenge (*n* = 10/group). Significance denoted as * *p* < 0.05, ** *p* < 0.01, *** *p* < 0.001, **** *p* < 0.0001.

Based on the neonatal mouse challenge model for HFMD, we further evaluated the antiviral protective ability of maternal antibodies (Figure 2A). Persistent weight monitoring of one-day-old neonatal mice revealed that, in comparison to the control and cross-protective groups, both the monovalent and trivalent inactivated virus immune groups exhibited significant weight gain (Figure 2A). Neonatal mice in the control and cross-protective groups progressively developed weight imbalance after 2 dpi. Subsequent analysis of clinical scores and survival curves revealed that neonatal mice in these groups displayed typical symptoms by 2 dpi, which worsened significantly over time, ultimately resulting in complete mortality within 10 days (Figure 2B,C). Notably, there was no significant difference in weight gain between the monovalent and trivalent inactivated virus immune groups. However, distinct protective effects against CA16 and CA10 were observed from the perspectives of clinical symptoms and mortality rates (Figure 2A–C). Specifically, upon the EV71 challenge, there was no notable difference in clinical symptoms between the EV71 monovalent inactivated virus immune group and the trivalent inactivated virus immune group, both achieving a consistent 70% survival rate. Conversely, during CA16 challenge, the CA16 monovalent inactivated virus immune group displayed no significant clinical symptoms and achieved a 100% survival rate, a result significantly different from that of the trivalent inactivated virus immune group (60% survival rate). Similarly, in the CA10 challenge, the survival rate was 70% in the CA10 monovalent inactivated virus immune group and 50% in the trivalent inactivated virus immune group, indicating significant differences in clinical symptoms and survival rates between the two groups. Overall, while the trivalent inactivated virus immune group exhibited weaker protection against CA16 and CA10 compared to the monovalent inactivated virus immune group, it is noteworthy that, when compared to the control and cross-protective groups, the trivalent inactivated virus immune group demonstrated substantial protective effects.

**Figure 3:**
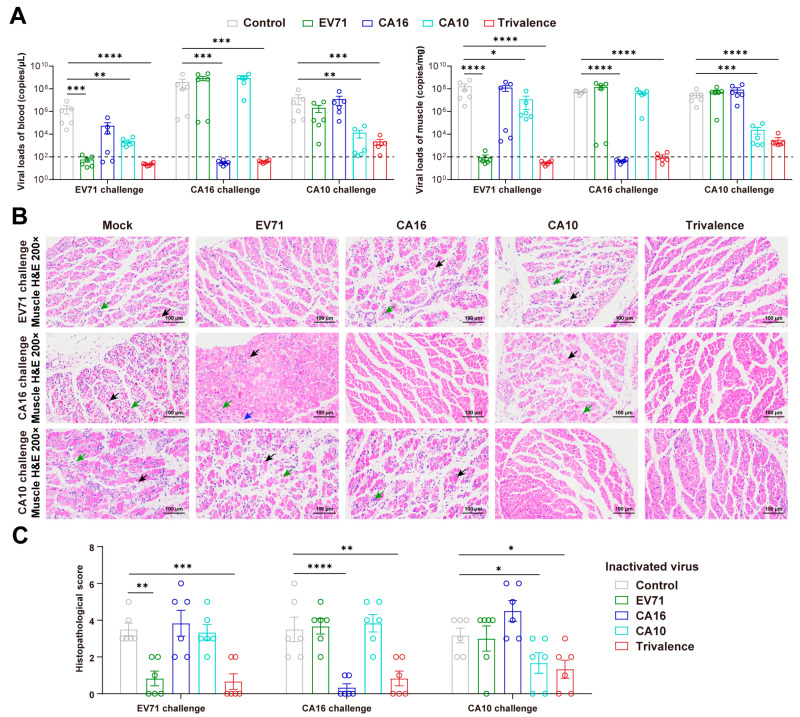
Virological and pathological assessment of feeding offspring mice after HFMD challenge. (**A**) Viral load determination in blood (**left**) and skeletal muscle (**right**) of neonatal mice (*n* = 6/group) at 5 dpi. Detection line marked with a dashed line. (**B**) Muscle cells undergo necrosis, with nuclear condensation, fragmentation, and loss of normal structure, while connective tissue proliferates for repair (black arrows). Granulocytes and lymphocytes infiltrate (green arrows). Muscle cells atrophy, reducing in size (blue arrows). Scale bar = 100 μm. (**C**) Comprehensive scoring of skeletal muscle tissue damage. Significance denoted as * *p* < 0.05, ** *p* < 0.01, *** *p* < 0.001, **** *p* < 0.0001.

Previous studies have reported that the typical symptom of the HFMD neonatal mouse challenge model is hind limb paralysis [48,49,50]. Therefore, the blood and muscle of infected mice at 5 days post-infection (dpi) were collected and subjected to virological and pathological assessments. As shown in Figure 3A, the viral load in the blood and muscle confirmed virus replication in the specific monovalent vaccine-treated mice (blood, *p* = 0.0002 for EV71 challenge, *p* = 0.0002 for CA16 challenge, *p* = 0.0024 for CA10 challenge; muscle, *p* < 0.0001 for EV71 challenge, *p* < 0.0001 for CA16 challenge, *p* = 0.0009 for CA10 challenge) and the trivalent vaccine-treated mice (blood, *p* < 0.0001 for EV71 challenge, *p* = 0.0002 for CA16 challenge, *p* = 0.0001 for CA10 challenge; muscle, *p* < 0.0001 for EV71 challenge, *p* < 0.0001 for CA16 challenge, *p* < 0.0001 for CA10 challenge). The viral load in blood and muscle was significantly lower in the inactivated virus-immunized group compared with the control group. Furthermore, as shown by HE and pathological comprehensive scores (Figure 3B,C), significant pathological damage was significantly alleviated in the specific monovalent (*p* = 0.0011 for EV71 challenge, *p* < 0.0001 for CA16 challenge, *p* = 0.0446 for CA10 challenge) and trivalent (*p* = 0.0005 for EV71 challenge, *p* = 0.0011 for CA16 challenge, *p* = 0.0371 for CA10 challenge) groups. The muscle of the inactivated virus vaccination group had an organized arrangement of skeletal muscle bundles and clear boundaries, but that of the control and cross-protection groups showed varying degrees of damage, characterized by a large amount of necrosis and dissolution of typical skeletal muscle cells, accompanied by infiltration of lymphocytes and granulocytes and a small amount of connective tissue hyperplasia. The above results collectively indicated that the monovalent vaccine failed to provide cross-protection against the various HFMD pathogens. Fortunately, passive immunity conferred by maternal antibodies induced by multivalent inactivated viruses protected neonatal mice from the lethal challenge of different HFMD pathogens.

### 3.3. Immunization of Cows with Trivalent Inactivated Viruses Produces Broad-Spectrum Neutralizing Antibodies

**Figure 4:**
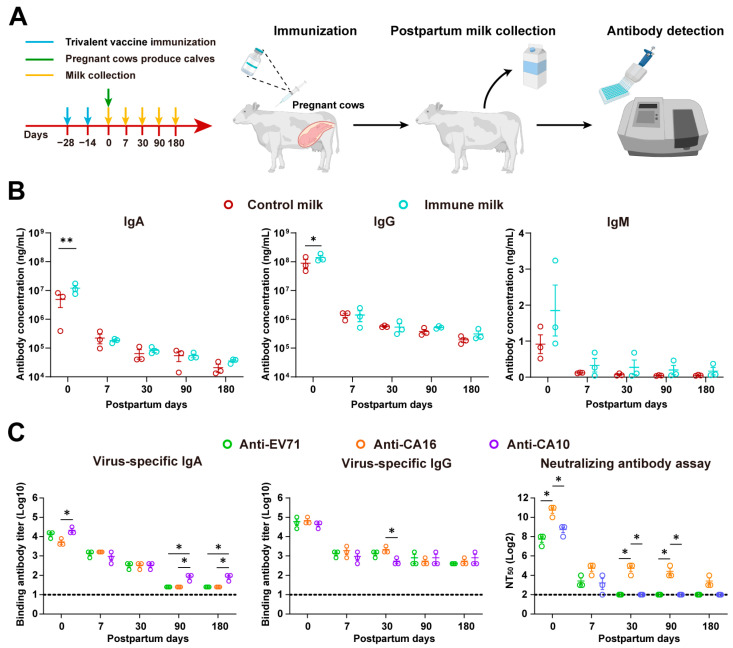
Antibody levels in milk of cows immunized with HFMD trivalent inactivated virus. (**A**) Schematic of pregnant cows immunized with HFMD trivalent inactivated virus and milk collection (*n* = 3/group). (**B**) Determination of IgA, IgG, and IgM antibody levels in control and immune milk on different postpartum days. (**C**) Quantification of HFMD virus-specific IgA/IgG antibodies and neutralizing antibodies in control and immune milk on different postpartum days. Significance denoted as * *p* < 0.05, ** *p* < 0.01.

The decline in maternal antibodies under basal conditions after six months increases the risk of newborns being re-exposed to HFMD pathogens, so novel strategies in addition to frequent vaccination are needed to compensate for the deficiency of neutralizing antibodies in children under 6 years of age [37]. Therefore, we propose an oral administration strategy of multivalent vaccine-immunized milk for preventing HFMD, which may serve as a supplement to vaccine prevention of HFMD (Figure 4A). Pregnant cows were immunized with EV71, CA16, and CA10 trivalent inactivated viruses, and the titers of IgG and IgA in milk collected at different postpartum stages were higher, especially in colostrum, while the IgM titer was lower (Figure 4B). Moreover, all antibody levels decreased rapidly within 7 days and then stabilized over time. No significant difference in antibody titers against EV71, CA16, and CA10 viruses was detected, indicating that similar vaccination effects were achieved for the three viruses, and colostrum and seven-day milk contained the highest titers of neutralizing antibodies against the three viruses (Figure 4C). Meanwhile, neutralizing antibodies against CA16 are higher and maintain for a longer period of time.

### 3.4. Immune Milk Provides Cross Protection against HFMD in Neonatal Mouse Models

**Figure 5:**
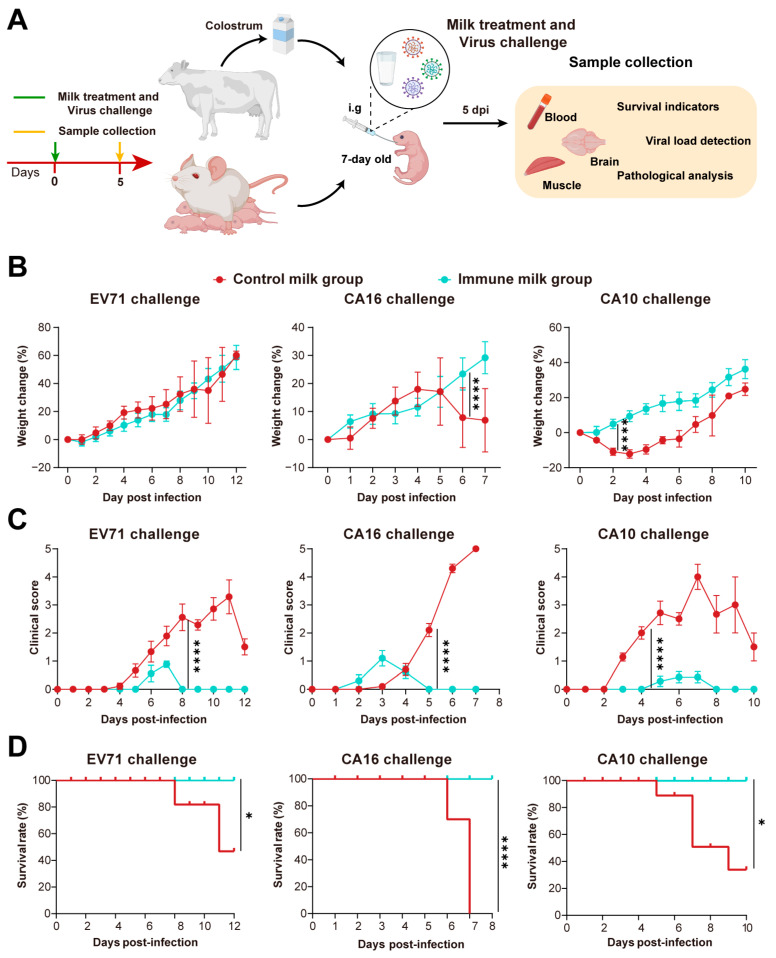
Protective effect of immune colostrum against HFMD challenge in neonatal mice. (**A**) Experimental setup for evaluating the protective effect of immune colostrum on neonatal mice challenged with HFMD. Seven-day-old neonatal mice (*n* = 6–10) were gavage-immunized with bovine milk to study preventive and protective effects against EV71/CA16/CA10 viruses. (**B**) Weight changes in seven-day-old neonatal mice infected with virus/colostrum mixture. (**C**) Clinical symptom scores in neonatal mice. (**D**) Survival rate of seven-day-old neonatal mice post HFMD challenge (*n* = 6). Significance denoted as * *p* < 0.05, **, **** *p* < 0.0001.

Based on HFMD challenge neonatal model mice, we further evaluated the cross protection of colostrum (Figure 5A). Continuous monitoring of weight changes and clinical symptom scores confirmed that after challenging neonatal mice with 100 LD_50_ of EV71, CA16, and CA10, immune milk treatment significantly reduced clinical symptoms and inhibited weight loss in neonatal mice infected with EV71, CA16, and CA10 (Figure 5B,C). Furthermore, immune milk treatment could completely protect them from death, while the mortality rates of mice treated with control milk were 55.55% for EV71, 100% for CA16, and 71.42% for CA10 (Figure 5D).

**Figure 6:**
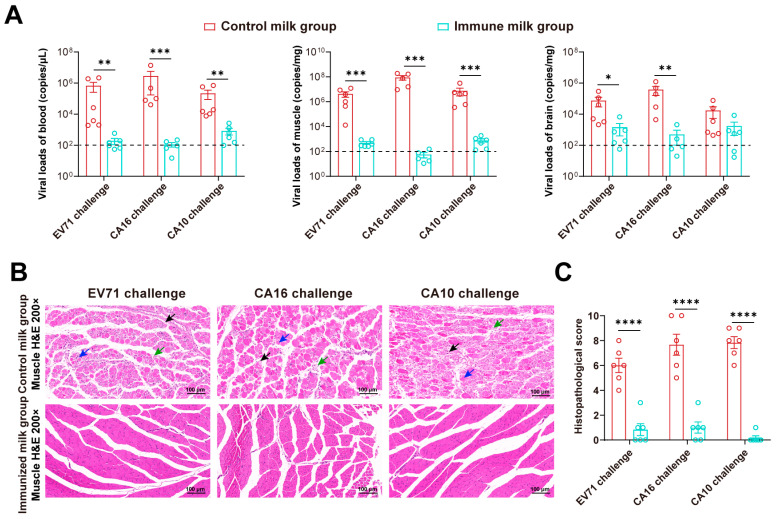
Virological and pathological assessment of various tissues in neonatal mice after gavage of HFMD viruses and colostrum. (**A**) Viral load determination in blood, skeletal muscle, and brain of neonatal mice (*n* = 6/group) at 5 dpi. Detection line marked with a dashed line. (**B**) Pathological analysis of skeletal muscle at 5 dpi. Muscle cells undergo necrosis, with nuclear condensation, fragmentation, and loss of normal structure, while connective tissue proliferates for repair (black arrows). Granulocytes and lymphocytes infiltrate (green arrows). Muscle cells atrophy, reducing in size (blue arrows). Scale bar = 100 μm. (**C**) Comprehensive scoring of skeletal muscle tissue damage. Significance denoted as * *p* < 0.05, ** *p* < 0.01, *** *p* < 0.001, **** *p* < 0.0001.

Moreover, compared with control milk, immune milk treatment significantly reduced virus levels in the blood (*p* = 0.0032 for EV71 challenge, *p* = 0.0008 for CA16 challenge, *p* = 0.0046 for CA10 challenge), muscle (*p* = 0.0005 for EV71 challenge, *p* = 0.0002 for CA16 challenge, *p* = 0.0004 for CA10 challenge), and brain (*p* = 0.0198 for EV71 challenge, *p* = 0.0061 for CA16 challenge, *p* = 0.0605 for CA10 challenge) (Figure 6A). Pathological observation and comprehensive pathological scoring also confirmed that immune milk ameliorated severe skeletal muscle damage compared to control milk, and no obvious muscle cell atrophy, necrosis, or granulocyte infiltration was observed (*p* < 0.0001 for EV71 challenge, *p* < 0.0001 for CA16 challenge, *p* < 0.0001 for CA10 challenge) (Figure 6B,C). These results collectively indicate that trivalent immune milk can provide significant broad-spectrum protection against three types of HFMD viruses in newborn mice, further confirming the potential of immune milk as passive immunity against various HFMD pathogens.

## 4. Discussion

There are many kinds of enteroviruses that cause HFMD outbreaks, including more than 36 serotypes such as EV71, CA16, CA6, CA10, and sporadic types A2, A4, A5, A8, A12, A14, and CV-B1-B5 [32,34,51,52,53,54,55,56,57]. Although EV71 vaccines marketed in Asia have shown resistance to most EV71 variants [58,59,60], they have not had a significant impact on the overall prevalence of HFMD [21,23,61], primarily due to changes in serology and genotype [31,62,63,64]. Although the development of multivalent HFMD vaccines can effectively prevent existing epidemic strains, it is difficult to effectively reduce the overall prevalence of HFMD and prevent the emergence of new strains and genotypes [20]. The prevalence of new strains and genotypes also challenges the protection rate of vaccination [20]. In addition, vaccine immunity has virus specificity and timeliness characteristics, requiring two or three booster doses to extend protection to 2 years [65,66]. The recurrence of HFMD is also clinically common, although most cases are attributed to enteroviruses of different serotypes [17], but the reason for weakened immunity cannot be ignored [17,59]. Breastfeeding provides infants with maternal antibodies that have been repeatedly shown to interfere with vaccine efficacy, potentially leading to reduced immunity [67,68]. It is also difficult to induce sufficient immune responses in children with a low vaccine response, immature immunity, or even immunodeficiency [24,25,26,27].

Research indicates that maternal antibodies can confer protection against EV71 for newborns for up to six months [37]. Amidst the COVID-19 pandemic, breast milk has been found to provide infants with passive immunity against SARS-CoV-2 [69]. This study further establishes that maternal antibodies, acquired by immunizing mother mice with multivalent vaccines against EV71, CA16, and CA10, can partially shield newborn mice from the three HFMD viruses. Similar findings have been reported in other studies [70,71]. In this study, while maternal antibodies from trivalent inactivated virus immunization did not fully protect neonatal mice and were slightly less effective against CA16 and CA10 compared to the monovalent inactivated virus immune group, they still demonstrated significant protective effects compared to the control and cross-protective groups. This underscores the challenge monovalent vaccines face in providing cross-protection against multiple viruses and highlights the importance of multivalent vaccines. However, the pace of vaccine updates and widespread vaccination may not match the rate of virus mutation and epidemiological patterns of the viruses. Therefore, HFMD, which is primarily transmitted through the gastrointestinal tract, might be prevented or treated with oral vaccines or drugs, potentially representing a new strategy similar to the efficacy of novel oral vaccines in preventing poliovirus [72]. During the COVID-19 pandemic, similar studies also confirmed the rationale and feasibility of oral immunization with bovine colostrum [43,44]. This study also demonstrated that the proposed polyvalent milk exhibited broad-spectrum neutralizing activity against various HFMD viruses and showed good preventive effects against CA10, CA16, and EV71 in a neonatal mouse model. Therefore, milk containing immunoglobulins against multiple HFMD viruses could potentially be developed as an optimal health product for the prevention or treatment of HFMD. Despite the effectiveness of polyvalent immune milk in animal models, its practical clinical application still faces challenges. Strict processing techniques are required to ensure the presence of passive immunity in milk, especially in specialized dairy products. Additionally, children allergic to milk may not benefit from consuming this polyvalent immune milk [73,74]. Furthermore, milk-based passive immunity is transient and cannot provide long-term protection similar to that of vaccination.

The landscape of intestinal infectious diseases is rife with numerous viruses and bacteria, posing a constant threat to the well-being of millions of children annually. The ever-present specter of pathogenic mutation and subtype variation poses a formidable challenge to vaccination-based preventive strategies. Furthermore, the potential risks associated with subjecting children to multiple vaccinations featuring new antigens remain largely unknown. This study provides and validates a supplementary protective paradigm to address variants outside of vaccines, utilizing multivalent immune milk and dairy products targeting emerging intestinal pathogens. This approach holds promise, particularly for non-allergic children, including those who are immunocompromised, as it offers a mechanism for acquiring transient immunity through milk consumption. This passive strategy presents a viable supplement to active vaccination methods.

## 5. Conclusions

This study demonstrates that maternal antibodies from immunized mother mice with multivalent HFMD vaccines can offer broad-spectrum passive protection against various HFMD strains in newborn mice, suggesting an alternative strategy beyond immune vaccines for preventing HFMD in infants and young children. Based on this, we further propose a multivalent milk protection strategy derived from multivalent vaccine immunization, showing potential as a passive immunization method for preventing HFMD in newborn mice. Despite existing shortcomings and the need for further clinical validation, this strategy could serve as a vaccine alternative for rapid response and timely control of emerging HFMD pathogens.

## Data Availability

The original contributions presented in the study are included in the article/Supplementary Materials. Further inquiries can be directed to the corresponding author.

## Supplementary Materials

The following supporting information can be downloaded at: https://www.mdpi.com/article/10.3390/vaccines12060570/s1, Table S1. Strains information of HFMD; Table S2. Scoring of clinical symptoms; Table S3. Primers and probes used in RT–PCR; Table S4. Scoring of tissue pathological damage.
