# Supplementary material for: The Milk of Cows Immunized with Trivalent Inactivated Vaccines Provides Broad-Spectrum Passive Protection against Hand, Foot, and Mouth Disease in Neonatal Mice
Source: Vaccines (Basel). 2024 May 23;12(6):570. doi: 10.3390/vaccines12060570 (PMC11209096; doi:10.3390/vaccines12060570)
Supplement: Supplementary file 1 [file vaccines-12-00570-s001.zip › vaccines-2991894-supplementary.pdf]

Supplemental Material

The Milk of Cows Immunized with Trivalent Inactivated Vaccines Provides Broad-Spectrum Passive Protection against Hand, Foot, and Mouth Disease in Neonatal Mice

Xiaohui Wei[1, †], Jing Wu[1, †], Wanjun Peng[1], Xin Chen[1], Lihong Zhang[1], Na Rong[1], Hekai Yang[1], Gengxin Zhang[1], Gaoying Zhang[2], Binbin Zhao[1, *] and Jiangning Liu[1, *]

1 NHC Key Laboratory of Human Disease Comparative Medicine, Beijing Key Laboratory for Animal Models of Emerging and Reemerging Infectious Diseases, Institute of Laboratory Animal Science, Chinese Academy of Medical Sciences and Comparative Medicine Center, Peking Union Medical College, Beijing 100021, China; xiaohui-wei@foxmail.com (X.W.)

2 Wuhan Servicebio Technology Co., Ltd., Wuhan 430079, China; gaoyingzhang@servicebio.cn

* Correspondence: zhaocaomei@163.com (B.Z.); liujn@cnilas.org (J.L.)

† These authors contributed equally to this work.

Table S1. Strains information of HFMD

Strains	GenBank	Source	Infective route	Infective dose
EV71	HQ712020.1	The mouse-adapted strain MP10 was cultured from clinically isolated EV71 strain FY0805 (GenBank: HQ882182)	i.g.	LD_{50}
CA16	EU262658.1	clinically isolated CA16 strain shzh05-1	i.g.	LD_{50}
CA10	MF688814.1	clinically isolated CA10 strain	i.g.	LD_{50}

Table S2. Scoring of clinical symptoms

Clinical score	Clinical symptoms
0	healthy
1	lethargy and inactivity
2	wasting
3	limb weakness
4	hind limb paralysis
5	moribundity and death

Table S3. Primers and probes used in RT–PCR

Virus	Primer name	Primer sequence
EV71	EV71-F	GCAGCCCAAAAGAACTTCACT
	EV71-R	TCTGCCACCCTATCTCCCT
	EV71-TaqProbe	FAM-TGCAAGGATGCTAGTGATATCCTGC-TAMRA
CA16	CA16-F	TAGGGACGCACGTGATCTGGGACTTCG
	CA16-R	GCACCAATGGGAACTACATAGTTAGTTTG
CA10	CA10-F	GAAATGGRGTGTTGGAAACCA
	CA10-R	TTTCTGCGRAGTTGGACAAAG
	CA10-TaqProbe	FAM- ATCAACCAYTTCTTCTCYCGCTCTGG-TAMRA

Table S4. Scoring of tissue pathological damage

Degree of pathological damage	Scoring of pathological damage					
	Denaturation	Necrosis	Inflammatory cell infiltration	Hyperplasia	Atrophy	Hemorrhage
Normal without damage	0	0	0	0	0	0
Very minor damage	1	1	1	1	1	1
Minor damage	2	2	2	2	2	2
Moderate damage	3	3	3	3	3	3
Severe damage	4	4	4	4	4	4
